# Morphological and clinical findings in Sri Lankan patients with chronic kidney disease of unknown cause (CKDu): Similarities and differences with Mesoamerican Nephropathy

**DOI:** 10.1371/journal.pone.0193056

**Published:** 2018-03-07

**Authors:** Julia Wijkström, Channa Jayasumana, Rajeewa Dassanayake, Nalin Priyawardane, Nimali Godakanda, Sisira Siribaddana, Anneli Ring, Kjell Hultenby, Magnus Söderberg, Carl-Gustaf Elinder, Annika Wernerson

**Affiliations:** 1 Division of Renal Medicine, Department of Clinical Science, Intervention and Technology, Karolinska Institutet, Stockholm, Sweden; 2 Department of Pharmacology, Faculty of Medicine, Rajarata University of Sri Lanka, Saliyapura, Sri Lanka; 3 Renal Unit, General Hospital, Polonnaruwa, Sri Lanka; 4 Faculty of Medicine, Rajarata University of Sri Lanka, Saliyapura, Sri Lanka; 5 Clinical Pathology and Cytology, Karolinska University Hospital, Stockholm, Sweden; 6 Division of CRC, Department of Laboratory Medicine, Karolinska Institutet, Stockholm, Sweden; 7 Pathology, Drug Safety and Metabolism, IMED Biotech Unit, AstraZeneca, Gothenburg, Sweden; Istituto Di Ricerche Farmacologiche Mario Negri, ITALY

## Abstract

In Sri Lanka, an endemic of chronic kidney disease of unknown origin (CKDu) is affecting rural communities. The endemic has similarities with Mesoamerican Nephropathy (MeN) in Central America, however it has not yet been clarified if the endemics are related diagnostic entities. We designed this study of kidney biopsies from patients with CKDu in Sri Lanka to compare with MeN morphology. Eleven patients with CKDu were recruited at the General Hospital, Polonnaruwa, using similar inclusion and exclusion criteria as our previous MeN studies. Inclusion criteria were 20–65 years of age and plasma creatinine 100–220 μmol/L. Exclusion criteria were diabetes mellitus, uncontrolled hypertension and albuminuria >1g/24h. Kidney biopsies, blood and urine samples were collected, and participants answered a questionnaire. Included participants were between 27–61 years of age and had a mean eGFR of 38±14 ml/min/1.73m^2^. Main findings in the biopsies were chronic glomerular and tubulointerstitial damage with glomerulosclerosis (8–75%), glomerular hypertrophy and mild to moderate tubulointerstitial changes. The morphology was more heterogeneous and interstitial inflammation and vascular changes were more common compared to our previous studies of MeN. In two patients the biopsies showed morphological signs of acute pyelonephritis but urine cultures were negative. Electrolyte disturbances with low levels of serum sodium, potassium, and/or magnesium were common. In the urine, only four patients displayed albuminuria, but many patients exhibited elevated α-1-microglobulin and magnesium levels. This is the first study reporting detailed biochemical and clinical data together with renal morphology, including electron microscopy, from Sri Lankan patients with CKDu. Our data show that there are many similarities in the biochemical and morphological profile of the CKDu endemics in Central America and Sri Lanka, supporting a common etiology. However, there are differences, such as a more mixed morphology, more interstitial inflammation and vascular changes in Sri Lankan patients.

## Introduction

Chronic kidney disease (CKD) is a global health problem. The leading causes of CKD are diabetes mellitus, hypertension, and glomerulonephritis [1], but chronic kidney disease of unknown cause (CKDu) is an emerging health problem in some low- and middle income countries, including El Salvador, Nicaragua, Egypt, Sri Lanka and India [2]. These endemics of CKDu share some mutual features; they affect rural populations, men are more often affected than women, and the countries have a hot climate. In many patients, CKDu will progress to end-stage renal disease, which might be catastrophic for individuals in poor areas and communities where access to renal replacement therapy often is limited or not affordable [3].

In Central America, the CKDu endemic is called Mesoamerican Nephropathy (MeN), and men working with heavy physical labor in warm areas near the Pacific Ocean often develop the disease [4, 5]. Clinically, the patients display elevated serum creatinine levels, normal or mild albuminuria, and inactive urine sediment. Members of our research team have previously studied the renal morphology and biochemical characteristics in two MeN cohorts in Nicaragua and El Salvador. We found a unique morphology with glomerulosclerosis, signs of glomerular ischemia and podocytic changes in combination with mild to moderate tubulointerstitial damage [6, 7]. The cause of MeN is not yet fully understood, but work-related heat stress with losses of water and salts is an established risk factor [8].

Kidney disease was the seventh most common cause of death and an increasing cause of death in Sri Lanka in 2012 according to the WHO [9]. In the last decades, a high prevalence of CKDu has been reported from rural areas [10–12]. Athuraliya et al (2009) published a retrospective study of 492 patients from two nephrology units, and they found that a high percentage of the patients (54% and 82%, respectively) had CKDu [10], i.e. CKD not associated with diabetes mellitus, hypertension, or other known kidney disease. The warm and dry North Central Province is one of the most heavily affected regions, and cross-sectional studies using dipstick albuminuria as a first screening report a prevalence of CKDu between 4–21% in this region [11, 13, 14]. The disease is more common in males [15, 16], and risk factors associated with CKDu are being a farmer [11, 13, 16, 17], using pesticides [16, 17], and having a family history of CKD [11, 15, 17].

Most studies on CKDu in Sri Lanka have used dipstick albuminuria as a screening method, and subjects with albuminuria have been further analyzed with serum creatinine. Patients with CKDu often have low-range albuminuria (<1g/24h) [10, 11, 18], and in addition the patients often have increased levels of urinary tubular injury markers [18, 19].

The renal morphology of CKDu in Sri Lanka has been reported in several studies [11, 20–23], and the morphology is often described as a tubulointerstitial nephritis/tubulointerstitial disease, but significant glomerular lesions such as global glomerulosclerosis, signs of glomerular ischemia and sporadic findings of focal glomerulosclerosis have also been reported [20, 23].

The etiology behind CKDu in Sri Lanka has not yet been established, but researchers have proposed a wide range of different environmental exposures, such as pesticides, herbicides, fertilizers, heavy metals, water hardness [12, 24, 25], and infections [26], as possible causes. Heavy metal exposure, by measuring levels in urine, has been evaluated in some studies, but with varying results [13, 24, 27].

There are many similar features of the CKDu endemics in Central America and Sri Lanka, but no proper comparative study of the renal morphology and clinical characteristics has, as of yet, been made. To compare available studies from the two regions is complicated due to differences in study design, semi-quantification of morphology findings, etc. Thus, to make a detailed comparison of the endemics in Sri Lanka and Central America possible, we designed this study of renal biopsies, biochemical and clinical characteristics from patients with CKDu in Sri Lanka using similar inclusion/exclusion criteria and study design as in our previous studies of MeN in Central America [6, 7].

## Methods

### Patients

Patients planned for kidney biopsy at Polonnaruwa General Hospital were recruited. Inclusion criteria were CKD of unknown cause, 20–65 years of age, and serum creatinine 100–220 μmol/L or eGFR 30–80 ml/min/1.73m^2^. Exclusion criteria were diabetes mellitus (fasting blood glucose >7 mmol/L), uncontrolled hypertension (>140/90 mmHg or more than one hypertensive drug prescription), proteinuria >1g/24h, or other known renal disease.

### Study procedure overview

The study was performed during one week in May/June at Polonnaruwa General Hospital. Blood pressure was tested the day before and on the day of the biopsy. At the morning of the kidney biopsy, blood and urine tests were collected from fasting participants (no intravenous fluids). Ultrasound was performed before and during the kidney biopsy. Participants were interviewed and a questionnaire was filled in. All participants stayed for a 24-hour observation after the biopsy procedure. Within one week, collected samples were transported to Karolinska University Hospital (KUH) for analysis.

### Kidney biopsies

Ultrasound-guided percutaneous kidney biopsies were performed using a spring-loaded biopsy needle (16 gauge, Bard Magnum, Bard Biopsy Systems, USA). The biopsies were divided and placed in: 4% buffered formaldehyde, Zeus fixative solution, and 2% glutaraldehyde + 1% paraformaldehyde in 0.1 M phosphate buffer, respectively. Within one week the biopsies were transported to KUH. At KUH, the formaldehyde fixed tissue was embedded in paraffin, sectioned and stained with Ladewig, periodic acid Schiff (PAS), hematoxylin-eosin (HE), and periodic acid silver methenamine (PASM). The tissue in Zeus solution was rinsed and snap frozen, and cryosections were incubated with antibodies against immunoglobulins (IgG, IgA, and IgM), C1q, C3, light chains kappa and lambda, and fibrinogen. Sections from the paraffin-embedded tissue and cryosections were evaluated with polarized light to detect any birefringent crystals. The tissue for transmission electron microscopy (2% glutaraldehyde) was embedded in epoxy resin and then cut into 60 nm thin sections. Histological evaluation with light microscopy, immunofluorescence, and electron microscopy was performed separately by two senior consultants in renal pathology (AW and MS) who thereafter discussed the results to reach consensus. Interstitial fibrosis, interstitial inflammation, and tubular atrophy in the cortical area were semi-quantified similar to Banff criteria [28], as follows: mild, affecting 6%-25%; moderate, affecting 26%-50%; and severe, affecting >50%. Glomerular hypertrophy and vascular pathology were semi-quantified as follows: no/normal, mild, moderate, or severe. When grading podocyte foot-process effacement, the number of slits per micron of glomerular basement membrane (GBM) in five random capillaries per glomerulus was calculated. Effacement was defined as widespread if ≥80% of the measured areas had <1.0 slit/μm GBM and segmental if 21–79% of the evaluated areas had <1.0 slit/μm GBM.

### Blood samples

The day before the kidney biopsy procedure, blood samples were analyzed for: RBC, WBC, platelets, INR, PT/aPTT, and/or bleeding time/clotting time at the laboratory of General Hospital Polonnaruwa. Just before the biopsy, new blood samples were collected, centrifuged, and the supernatant was transferred to transport vials stored at -40°C until transport on dry ice to KUH. At Karolinska University Hospital laboratory (KUHL), the samples were analyzed according to standard protocols for glucose, CRP, creatinine (IDMS traceable), BUN, sodium, potassium, magnesium, calcium, phosphate, albumin, alanine aminotransferase, uric acid, cystatin C (IFCC traceable), beta-2-microglobulin, aldosterone, renin, complement (C1q, C3, C3d, C4), antinuclear antibody, and anti-neutrophil cytoplasmic antibody screening, anti-glomerular basement membrane antibodies, and screening for hepatitis B and C and HIV. eGFR was calculated using the CKD-EPI equation for creatinine [29] and creatinine+cystatin C [30]. Immunofluorescence assays were used to detect Hantaan virus IgG and Puumalavirus IgG antibodies. ReaScan^®^ Dobrava-Hantaan IgM test (Reagena, Toivala, Finland) was used to detect Dobrava and Hantaan virus IgM antibodies. ELISA was used to detect Puumula virus IgM and leptospirosis IgM. Samples positive on ELISA for leptospirosis IgM were send for confirmation with microscopic agglutination test testing for 15 different leptospira serovars at SSI Copenhagen, Denmark.

### Urine samples

At Polonnaruwa General Hospital, urine sediment and urine culture were analyzed before the biopsy in all but two patients (missing sediment) and three patients (missing urine culture) due to a logistic error. The missing samples were collected and analyzed 5–6 months later. At the morning of the biopsy, new samples were collected and stored at -40°C until shipment on dry ice to KUH. At KUHL, the urine was analyzed according to standard protocols for uric acid, sodium, potassium, magnesium, creatinine, albumin, α-1-microglobulin, and neutrophil gelatinase-associated lipocalin. Urine heavy metal (As, Cd, Hg, Pb, U, and V) analysis was performed by ALS Scandinavia AB (Luleå, Sweden) using inductively coupled plasma mass spectrometry [31]. KIM-1 was measured using a solid-phase sandwich ELISA assay (R&D Systems, Minneapolis, USA). Fractional excretion of sodium (FENa) was calculated as: (urinary sodium x serum creatinine)/(serum sodium x urinary creatinine). Fractional excretion of potassium (FEK) was calculated using the same equation. Fractional excretion of magnesium (FEMg) was calculated as: (urinary magnesium x serum creatinine)/((0.7 x serum magnesium) x urinary creatinine). Reference range urine heavy metals: Arsenic <100 μg/L [32]. Cadmium-creatinine ratio <2 μg/g [33, 34]. Mercury-creatinine ratio <50 μg/g [33]. Lead <4 μg/L [35, 36]. Uranium (US geometric mean levels) 0.005–0.01 μg/L [37]. Vanadium normal levels ~0.5 μg/L [38].

### Questionnaires

A questionnaire was completed by interview of the participants (by CJ). The questionnaire contained open and closed-ended questions concerning medical history (current medicines, use of analgesics, chronic illnesses, snake bites, family history of CKD), work history (current and past work tasks, type of crops cultivated, days working in field per year), work-related chemical exposure (pesticides, herbicides, fertilizers), liquid intake, water source, and smoking habits.

### Ethical statement

The study received ethical clearance from the ethics review committee at the Faculty of Medicine & Allied Sciences, Rajarata University of Sri Lanka, Saliyapura (ERC/2016/06) and the regional ethical review board in Stockholm (Dnr 2012/441-31/3; 2013/1225-32; 2015/849-32; 2016/747-32). All participants gave written informed consent.

## Results

Thirteen patients were recruited to the study. Two patients were excluded because they did not fit the inclusion criteria, one patient due to high fasting blood glucose (16.7 mmol/L) and one patient due to more than one blood pressure medication. In total 11 patients with a mean age of 48 ± 11 years and a mean eGFR of 38 ± 14 ml/min/1.73m^2^ were included in the study.

### Clinical data and questionnaire

Clinical and questionnaire data are presented in Table 1. All included participants were or had been rice paddy farmers, sometimes in combination with tobacco and vegetable farming. Two participants also worked outside of their own field (one agricultural worker and one pesticide applicator). The mean liquid intake was 4.3 ± 0.9 L/day; the liquid mostly consumed was water (74–97% of the intake), followed by tea (3–25%). Own well was the most common water source. Seven patients stored their water in plastic tanks, two patients in clay pots and two patients in aluminum pots. Medical history and medicine usage are presented in Table 2. No patient used nonsteroidal anti-inflammatory drugs or aspirin. Only three patients used acetaminophen regularly, 2.5 g and 2 g per week and 1 g every other week respectively. Seven participants reported prior use of Ayurvedic medicines, with a mean duration of 0.5 (range 0.08–2) years. All patients reported chemical exposure to pesticides and fertilizers (S1 Table).

**Table 1:** Patient characteristics and questionnaire results of working history, n = 11.

**Age (years)**	48 ± 11 (27–61)
**Weight (kg)**	56.6 ± 8.8 (46.9–77.0)
**Height (m)**	1.66 ± 0.07
**BMI (kg/m**^**2**^**)**	20 ± 3 (16–28)
**Systolic BP (mmHg)**	122 ± 20 (97–160)^a^
**Diastolic BP (mmHg)**	78 ± 9 (58–90)
**Kidney length (cm)**	8.7 ± 0.6 (7.8–9.8)
**Family history of CKD**	3 (27%)
**Kidney stones**	0 (0%)
**Snake bites**	1 (9%)
**Current occupation**	
Farmer	7 (64%)
Farmer and construction worker	1 (9%)
Farmer, agricultural worker and rice mill laborer	1 (9%)
Farmer and pesticide sprayer	1 (9%)
Security officer	1 (9%)
**Total farming years**	24 ± 13 (3–40)
**Days working in field/year**	37 ± 19 (20–80)
**Total days working in field**	807 ± 573 (174–2000)
**Smoking years**	11 ± 12 (0–30)
**Pack years**	1.6 ± 1.9 (0–6.2)
**Liquid intake/day (L)**	4.3 ± 0.9 (3.0–6.0)
**Main source of water last five years**	
Own well	6 (55%)
Village well	2 (18%)
Tube well	2 (18%)
Main supply	1 (9%)

*Note*: Results are presented as mean ± SD (range) or cases (percentage).

^a^ Two patients had increased systolic blood pressure at the morning of the biopsy (145/90 and 160/80 respectively), but were normal the day before the biopsy (130/90 and 130/80 respectively).

**Table 2:** Questionnaire results of chronic diseases and medicine use.

Study no	Chronic diseases	Drug 1	Drug 2	Drug 3	Drug 4	Drug 5	Drug 6	Drug 7
1	Pain in legs	KCl 600 mg, 3/d.	CaCO_3_ 500 mg, 1/d.	Gabapentin 300 mg, 1/d.	Fluoxetin 20 mg, 1/d.	Amitriptyline 12.5 mg, 1/d.		
2	Knee pain	-						
3	Asthma	Alfacalcidol 0.25 μg, 1/d.	Omeprazol 20 mg, 2/d.	Famotidine 20 mg, 2/d.	Domperidone 10 mg, 1/d.	Gabapentin 100 mg, 1/d.	Salbutamol Inh 400 μg.	Beclamethasone Inh 200 μg.
4	Hypertension	Losartan 25 mg, 2/d.	CaCO_3_ 500 mg, 1/d.	FeSO4 200 mg, 2/d.				
5	-	Losartan 12.5 mg, 1/d.	CaCO_3_ 500 mg, 1/d.					
6	-	-						
7	Joint pain	CaCO_3_ 250 mg, 1/d.						
8	Insomnia	Alfacalcidol 0.25 μg, 1/d.	CaCO_3_ 500 mg, 1/d.	FeSO4 200 mg, 1/d.	Amitriptyline 12.5 mg, 1/d.			
9	-	CaCO_3_ 500 mg, 1/d.						
10	-	-						
11	Hemorrhoids	Spironolactone 12.5 mg, 1/d.	KCl 600 mg, 1/d.	CaCO_3_ 500 mg, 1/d.	Alfacalcidol 0,25 μg, 1/d.			

*Note*: Abbreviations: KCl, Potassium chloride. CaCO_3_, Calcium carbonate. FeSO4, Iron (II) sulfate. Inh, inhalation.

### Blood test results

Most blood test results are presented in Table 3. CRP was <3 mg/L in all but one patient (Patient 8, 44 mg/L). Four patients had slightly increased complement C4 (0.33, 0.37, 0.43 and 0.46 g/L), which could indicate inflammation, remaining complement levels were normal. No anti-GBM or anti-neutrophil cytoplasmic antibodies were found. Immunofluorescence was positive speckled for antinuclear antibodies in three patients, subsequent analysis for double-stranded DNA, centromere antibodies, and extractable nuclear antigens were negative. Hepatitis B and C, and HIV screening were all negative. Hantaan virus serology was positive for IgG in eight patients and negative for IgM in all patients. Puumala virus was negative for IgG and IgM in all patients. Leptospirosis IgM (ELISA) were positive in four patients, but subsequent analysis with microscopic agglutination test were negative.

**Table 3:** Serum test results, n = 11.

Serum variable	Mean ± SD (range)
Creatinine (μmol/L)	184 ± 50 (120–267)
Cystatin C (mg/L)	1.96 ± 0.53 (1.18–2.90)
eGFR_Cr_ (ml/min/1.73m^2^)	40 ± 14 (21–70)
eGFR_Cr+Cyst C_ (ml/min/1.73m^2^)	38 ± 14 (20–66)
Sodium (mmol/L)	140 ± 4 (134–147)^a^
Potassium (mmol/L)	4.3 ± 1.0 (2.2–6.3)^b^
Magnesium (mmol/L)	0.70 ± 0.16 (0.43–0.88)^c^
Uric Acid (μmol/L)	420 ± 97 (201–583)^d^
Calcium (mmol/L)	2.25 ± 0.40 (1.12–2.56)^e^
Albumin (g/L)	38 ± 7 (18–45)
Phosphate (mmol/L)	1.0 ± 0.2 (0.59–1.4)
Glucose (mmol/L)	5.2 ± 1.1 (2.9–6.8)
Hemoglobin (g/L)	124 ± 19 (100–156)
White blood cell count (10^9^/L)	7.35 ± 1.35 (4.91–10.25)
Aldosterone (nmol/L)	0.36 ± 0.27 (0.09–0.95)^f^
Renin (mIU/L)	28 ± 26 (3–90)^g^
Dobra-Hantaan virus IgM	All negative
Hantaan virus IgG	8 positive, 3 negative
Puumala virus IgM	All negative
Puumala virus IgG	All negative

Note:

^a^ Low sodium (<137 mmol/L) in one patient.

^b^ Low potassium (<3.5mmol/L) in two patients (2.2 and 3.4 mmol/L).

^c^ Low magnesium (<0.7 mmol/L) in four patients (0.43, 0.44, 0.61, and 0.64 mmol/L).

^d^ High uric acid (>416 μmol/L) in six patients.

^e^ Low calcium in two patients (1.12 and 2.07 mmol/L) and high calcium in two patients (2.55 and 2.56 mmol/L). The patient with calcium 1.12 mmol/L had serum albumin 18 g/L.

^f^ High aldosterone (>0.65 nmol/L) in two patients (0.77 and 0.95 nmol/L).

^g^ High renin (>40 mIU/L) in two patients (50 and 90 mIU/L).

### Urine test results

Urine test results are presented in Table 4. Urine dipstick showed 1+ albumin in two patients. In sediment, one patient had 8–10 erythrocytes per high-power field, all others had ≤5. All patients had <5 white blood cells per high-power field. Patient 1 had a few granular casts, no casts were detected in the other patients. No crystals were found. In the two patients with hypokalemia, urinary potassium was >20 mmol/L and FEK was 25% and 23% respectively, indicating renal potassium wasting [39, 40]. All patients had FEMg >4% indicating urinary magnesium losses [41]. Urine cadmium, mercury and lead levels were all non-toxic [33–35, 42–43]. Mean total arsenic was 42 μg/g creatinine, which is higher than in the US [44] but similar to findings in Korea [45], and all samples were below toxic levels (<100 μg/L) [32].

**Table 4:** Urine test results including urine heavy metals, n = 11.

Urine variable	Mean ± SD (range)
Culture	All negative
Creatinine (mmol/L)	8.4 ± 4.5 (2.4–17.2)
ACR (mg/mmol)	7.2 ± 14.1 (0.1–47.2)^a^
A1M-creatinine ratio (mg/mmol)	5.2 ± 4.1 (0.8–14.5)^b^
NGAL (μg/L)	47 ± 52 (17.5–154)^c^
KIM-1 (ng/ml)	1.1 ± 0.98 (0.19–3.44)
Sodium (mmol/L)	104 ± 71 (36–299)
FENa (%)	1.8 ± 0.8 (0.5–3.3)
Potassium (mmol/L)	27 ± 14 (5–50)
Potassium-creatinine ratio	3.3 ± 0.9 (1.9–5.3)
FEK (%)	15 ± 6 (8–25)
Magnesium (mmol/L)	2.1 ± 0.9 (1.1–3.7)
FEMg (%)	11 ± 5 (4–17)
Uric acid (mmol/L)	1.3 ± 0.6 (0.5–2.6)
Uric acid-creatinine ratio	0.17 ± 0.03 (0.12–0.23)
Arsenic (μg/L)	37 ± 21 (4.5–77)
Arsenic-creatinine ratio (μg/g)	42 ± 23 (17–98)
Cadmium (μg/L)	0.42 ± 0.30 (0.11–1.10)
Cadmium-creatinine ratio (μg/g)	0.49 ± 0.27 (0.2–1.05)
Mercury (μg/L)	0.24 ± 0.18 (<0.2–0.69)^d^
Mercury-creatinine ratio (μg/g)	0.3 ± 0.22 (0.08–0.79)
Lead (μg/L)	0.6 ± 0.5 (<0.5–1.72)^e^
Uranium (μg/L)	<0.02 μg/L in all patients
Vanadium (μg/L)	0.08 ± 0.06 (<0.1–0.22)^f^

*Note*: Abbreviations: ACR, albumin-creatinine ratio. A1M, alpha-1-microglobulin. NGAL, neutrophil gelatinase-associated lipocalin. KIM-1, kidney injury molecule-1. FENa, fractional excretion of sodium. FEK, fractional excretion of potassium. FEMg, fractional excretion of magnesium.

When calculating means, values below measurable levels were converted to half of the lowest measurable value: albumin <3.0 mg/L was converted to 1.5 mg/L; A1M <6 mg/L to 3 mg/L; NGAL <35 μg/L to 17.5 μg/L; mercury <0.2 μg/L to 0.1 μg/L; lead 0.5 μg/L to 0.25 μg/L; and vanadium <0.1 μg/L to 0.05 μg/L.

^a^ ACR was >3.4 mg/mmol in four patients at 4.9, 11.3, 13.2 and 47.3 mg/mmol.

^b^ A1M was > 0.7 mg/mmol in 10 patients. In one patient A1M was below measurable levels (<6 mg/L).

^c^ NGAL was >50 μg/L in three patients at 111, 115, and 154 μg/L.

^d^ Five patients had mercury below measurable levels, i.e. <0.2 μg/L.

^e^ Four patients had lead below measurable levels, i.e. <0.5 μg/L.

^f^ Eight patients had vanadium below measurable levels, i.e. <0.1 μg/L.

### Kidney biopsy morphology

Kidney biopsies were collected from all 11 patients. No adverse events were reported. The specimens contained 7–29 glomeruli (mean 17 glomeruli/specimen). Polarized light on paraffin-embedded tissues detected no crystals. Immunofluorescence showed no signs of immune complex disease. A summary of the light microscopy and electron microscopy findings are presented in Table 5.

**Table 5:** Summary of renal morphology findings by light and electron microscopy.

**Mean number of glomeruli**	17 ± 6 (7–29)	
***Light microscopy***		
**Glomerular changes, n = 11**	**Cases**	**Percentage**
Mean % globally sclerosed glomeruli	43 ± 20 (8–75) %	
% globally sclerosed glomeruli		
<25%	2	18%
25–50%	4	36%
>50%	5	45%
Segmental scleroses	0	0%
Glomerular hypertrophy		
0	0	0%
1	6	55%
2	4	36%
3	1	9%
Wrinkled GBM / Periglomerular fibrosis		
Yes	7	64%
No	4	36%
**Tubulointerstitial changes, n = 11**		
Tubular atrophy		
0	0	0%
1	10	91%
2	1	9%
3	0	0%
Interstitial fibrosis		
0	0	0%
1F	6	55%
2F	4	36%
3F	1	9%
Interstitial inflammation		
0	2	18%
1	5	45%
2	2	18%
3	2	18%
**Vascular changes, n = 10**		
Intimal thickening, n = 10		
0	5	50%
1	2	20%
2	3	30%
3	0	0%
Smooth muscle hyperplasia, n = 10		
0	6	60%
1	4	40%
2	0	0%
3	0	0%
Arteriolar hyalinosis, n = 11		
0	1	9%
1	7	64%
2	3	27%
3	0	0%
***Electron microscopy*, *n = 11***		
**GBM thickness (nm)**	307 ± 39 (259–349)	
**Podocyte foot processes (slits/μm GBM)**	1.5 ± 0.2 (1.2–1.8)	
**Podocyte foot process effacement**		
No (normal)	11	100%
Segmental effacement	2	18%
Widespread effacement	0	0%
**Endothelial cells**		
Normal	11	100%
Swollen	0	0%
**Podocyte cytoplasm inclusions**		
Yes	9	82%
No	2	18%
**Immune complex deposits**		
Yes	0	0%
No	11	100%

Results are presented as mean ± SD (range) or cases and percentage.

Abbreviations: GBM, glomerular basement membrane.

#### Light microscopy

**Fig 1:**
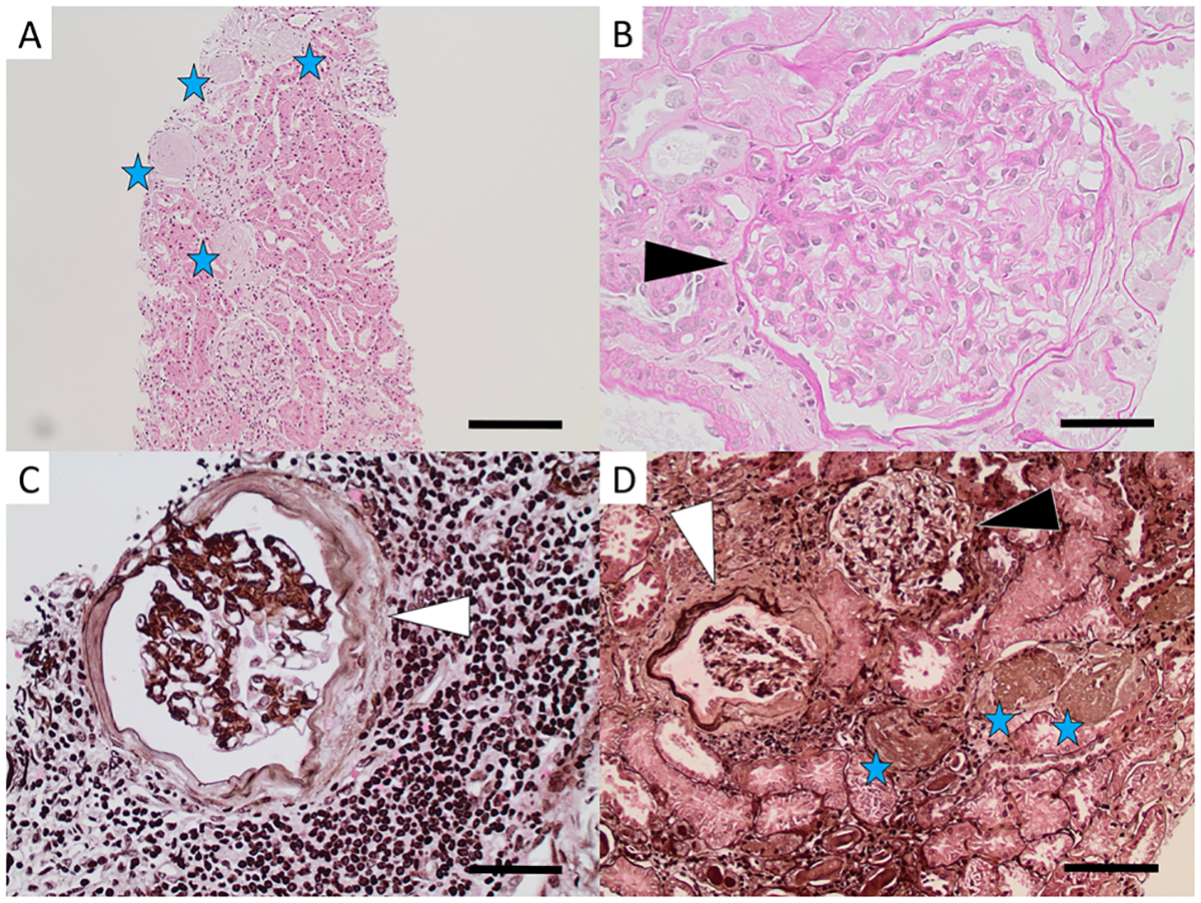
Light microscopy images of glomerular pathology in Sri Lankan patients with CKDu. Global glomerulosclerosis (stars in A and D) and glomerular hypertrophy (black arrow heads in B and D) of varying degree were found in all biopsies. Signs of glomerular ischemia with thickening of Bowman’s capsule (white arrow heads in C and D) and/or wrinkling of the capillaries was seen in seven patients. [Fig A: hematoxylin-eosin from Patient 4, bar = 200μm. Fig B: periodic acid Schiff from Patient 6, bar = 50μm. Fig C: periodic acid silver methenamine from Patient 3, bar = 50μm. Fig D: periodic acid silver methenamine from Patient 6, bar = 100μm.].

**Fig 2:**
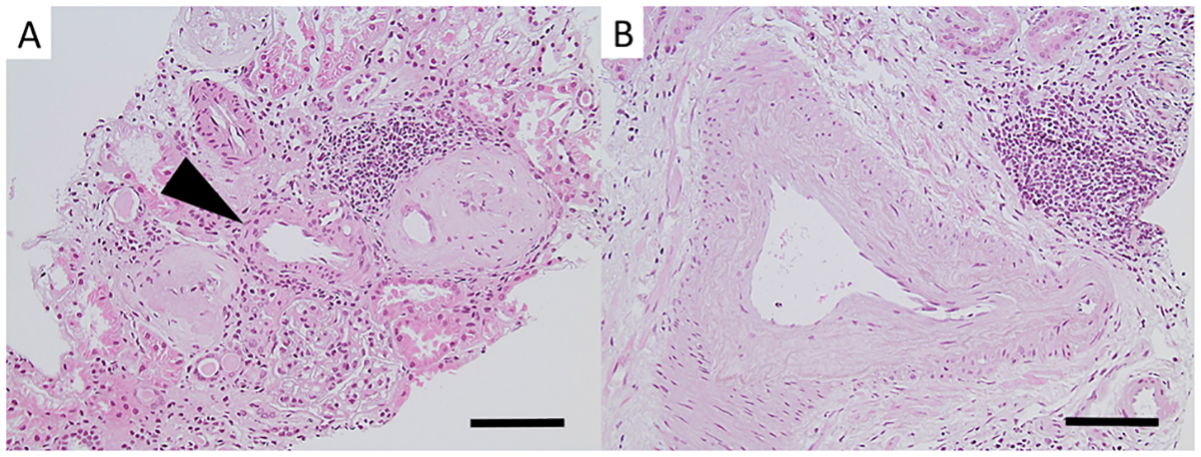
Light microscopy images of vascular pathology in Sri Lankan patients with CKDu. Most of the biopsies showed no or mild intimal fibrosis in arteries (arrow head in A). Three patients showed moderate intimal fibrosis (B). [Fig A: hematoxylin-eosin from Patient 6, bar = 100μm. Fig B: hematoxylin-eosin from Patient 3, bar = 100μm.].

**Fig 3:**
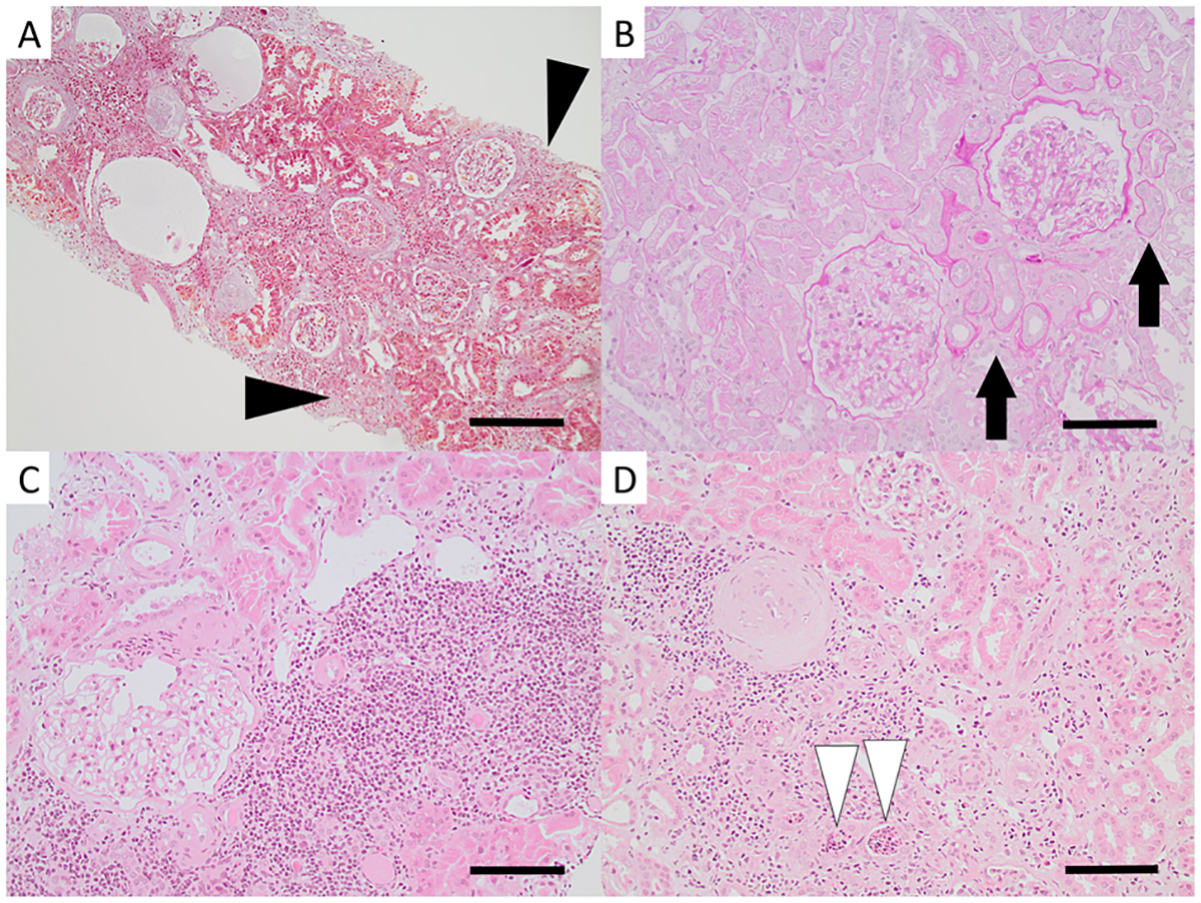
Light microscopy images of tubulointerstitial pathology in patients with CKDu in Sri Lanka. Mild to moderate interstitial fibrosis was found in most patients (black arrow heads in A). Tubular atrophy was mostly mild (black arrows in B). Interstitial inflammation was of varying degree ranging from none to severe (B, A, D and C). Signs of pyelonephritis with interstitial inflammation and neutrophil granulocytes in tubules were found in two patients (white arrow heads in D). [Fig A: Ladewig from Patient 7, bar = 200μm. Fig B: periodic acid Schiff from Patient 4, bar = 100μm. Fig C: hematoxylin-eosin from Patient 3, bar = 100μm. Fig D: hematoxylin-eosin from Patient 11, bar = 100μm.].

Glomerular pathology: Global glomerulosclerosis was found in all patients, mean 43% (range 8–75%) of all glomeruli (Fig 1A and 1D). All participants had glomerular hypertrophy, in most cases mild to moderate (Fig 1B and 1D). No segmental scleroses or endocapillary cell proliferation was found. Mild mesangial matrix increase was seen in all but two patients, and mild cell proliferation was found in one patient. In seven patients there were signs of glomerular ischemia with thickening of Bowman’s capsule and/or wrinkling of the GBM (Fig 1C and 1D), and in three of these cases there was a concurrent moderate intimal thickening in renal arteries (Fig 2B), indicating that the ischemic signs might be caused by vascular pathology. In the remaining four cases the vascular damage was only mild (Fig 2A). Cystic dilatation of Bowman’s capsule was seen in three patients (Patients 5, 7, and 8) (Fig 3A).

Tubular and interstitial pathology: Tubular atrophy was in most cases mild (Fig 3B). Tubulitis was seen in three patients (Patients 3, 10, and 11), and clusters of intratubular neutrophil granulocytes were found in Patients 10 and 11 (Fig 3D). Interstitial inflammation was of varying degree—two patients (Patients 1 and 9) had no inflammation, five patients had mild (Fig 3B), two patients (Patients 7 and 11) had moderate (Fig 3A and 3D), and two patients (Patients 3 and 10) had severe inflammation (Fig 3C). The inflammation consisted of mainly lymphocytes. Occasional eosinophils were found in Patient 3. Interstitial fibrosis was mostly mild to moderate (Fig 3A), but one patient (Patient 3) displayed severe interstitial fibrosis. The mild interstitial fibrosis was in all cases focal and the moderate interstitial fibrosis was in most cases diffuse.

Vascular pathology: Most specimens had no intimal thickening (Fig 2A), but five had mild to moderate thickening (Fig 2B). Mild smooth muscle hyperplasia was present in four biopsies. Mild to moderate arteriolar hyalinosis was found in all but one biopsy.

#### Electron microscopy

**Fig 4:**
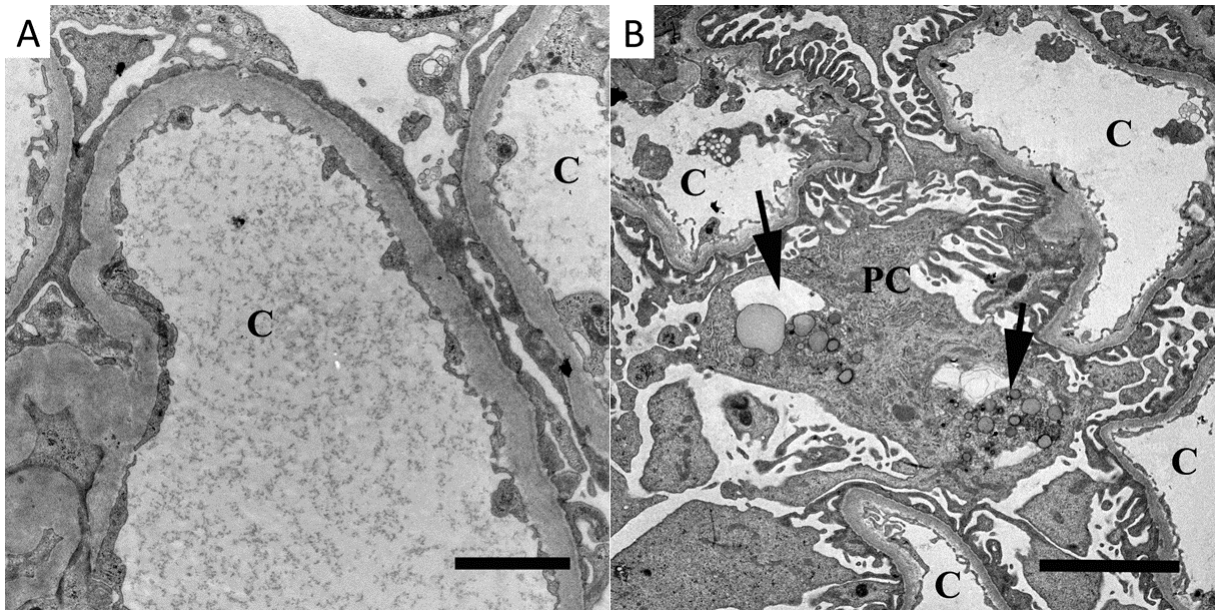
Transmission electron microscopy findings. Segmental podocytic foot process effacement was observed in two patients (A, Patient 4). Podocytic cytoplasm inclusions of vacuoles or lipofuscin-like-bodies (arrows in B, Patient 6) were found in the majority of the patients. c = capillary, pc = podocyte. Bars = (A) 2μm, (B) 5 μm.

No immune complex deposits were seen. Two patients showed segmental podocytic foot process effacement (Fig 4A). The majority of the patients had an increased amount of podocytic cytoplasm inclusions such as vacuoles and/or lipofuscin-like bodies (Fig 4B). Bowman’s space was normal in all patients. Some of the tubular cells showed reduced brush borders and reduced size.

#### Individual morphology results

In six patients (Patients 1, 2, 4, 6, 7, and 9) the main finding was glomerulosclerosis, glomerular hypertrophy, and mild to moderate tubulointerstitial changes. Two of the patients also had signs of glomerular ischemia (Patients 6 and 7).

In two patients (Patients 3 and 8) the main finding was chronic interstitial nephritis, with mild to moderate tubular atrophy, tubulitis (Patient 3), moderate to severe interstitial fibrosis, mild to severe interstitial inflammation, and moderate vascular changes.

Pyelonephritis was the main diagnosis in two patients (Patients 10 and 11), with moderate to severe interstitial inflammation, granulocytes in the tubular lumina, tubulitis, mild to moderate interstitial fibrosis, and mild vascular changes.

In one patient (Patient 8), the morphology indicated nephrosclerosis with moderate intimal thickening and mild smooth muscle hyperplasia of arteries, signs of glomerular ischemia, and mild to moderate tubulointerstitial changes.

## Discussion

In this study we have examined kidney biopsies and biochemical characteristics of 11 male farmers with CKDu in Sri Lanka. We have used similar inclusion and exclusion criteria and study design as in our previous studies of CKDu in Central America, thus enabling a detailed comparison of the two endemics.

The renal morphology in this study showed a more mixed pattern compared to our studies in Central America, where the morphology was relatively homogenous in all cases [6, 7]. Six cases displayed a MeN-like morphology with varying degree of glomerulosclerosis (29–54%), glomerular hypertrophy, mild to moderate tubulointerstitial changes, and mild vascular changes. However, signs of glomerular ischemia, a common finding in our previous cohorts, were only found in two of the six MeN-like cases. Further, no segmental sclerosis was found in this cohort, a finding that we have seen in a few patients in previous studies.

Of the remaining cases, two showed findings indicating active and chronic pyelonephritis with tubulitis, granulocytes found in the tubular lumina, and moderate to severe interstitial inflammation; two showed a morphological pattern of chronic tubulointerstitial nephritis with moderate to severe interstitial fibrosis and interstitial inflammation; and one showed nephrosclerosis with vascular changes, moderate intimal thickening, mild smooth muscle hypertrophy, and arteriolar hyalinosis.

From our results one can speculate whether these heterogeneous findings in the kidney biopsies represents different stages or severities of one disease or represent different diseases, with multiple etiologies. Nanayakkara et al described in their study of kidney biopsies from CKDu patients in Sri Lanka that the tubulointerstitial damage sometimes was accompanied by massive interstitial inflammation, and they concluded that this was the primary lesion [20]. In another study from Sri Lanka, acute and chronic tubulointerstitial lesions and glomerular scarring were found in patients with acute symptoms (backache, fatigue, joint pain, or dysuria) and kidney injury, indicating that there in some cases may be an acute disease prior to the development of CKD [22]. Although the patients in our study did not present with any acute symptoms, we saw severe interstitial mononuclear inflammation in two biopsies. We also considered if this could be a primary lesion and the other biopsies were later phases. Further studies are needed to elucidate this question. However, the two biopsies with severe inflammation also had chronic tubulointerstitial and vascular changes, indicating a more longstanding disease. One of the biopsies with severe interstitial inflammation was diagnosed morphologically as active and chronic pyelonephritis, but urine cultures were negative. One could speculate whether there could be an unknown pathogen, and we propose that infectious diseases as a cause behind CKDu should be further studied. In Central America, a recently published study from 11 sugarcane workers, with an acute inflammatory illness and kidney injury, reports findings of acute tubulointerstitial disease in kidney biopsies [46]. The authors suggests that the findings are early lesions in Mesoamerican Nephropathy/CKDu in Central America, which also supports that inflammatory and infectious etiologies need to be further investigated.

Due to the findings of interstitial inflammation and signs of pyelonephritis, we analyzed serum for Hantavirus and leptospirosis, common infections in rural Sri Lanka. Notably, elevated Hantavirus IgG-antibody levels, indicating a past infection, were found in 8 of 11 patients. IgM-antibody levels for Hantavirus and leptospirosis were negative. A recent publication from Sri Lanka showed that 55% of patients with CKDu were seropositive (IgG) for Hantavirus compared to 14% in controls but had no prior history of Hantavirus-associated illness [26]. Also in our study, none of the patients reported any hospitalization due to Hantavirus-associated symptoms, suggesting a subclinical infection. Whether a subclinical infection could result in renal scarring or a susceptibility to renal damage is to our knowledge not known; however, studies have reported a higher prevalence of antibodies against Hantavirus in other CKD populations compared to controls [47, 48]. We cannot rule out any prior leptospirosis infection in the present study since IgG levels were not measured. Concomitant infections with Hantavirus and leptospirosis has been reported to be common in Sri Lanka [49] and this calls for further studies in the CKDu population. Hantavirus and leptospirosis have been proposed as possible contributing factors in MeN [50], but data on Hantavirus infection in Central America are scarce [51] and no published data have, as of yet, supported Hantavirus or leptospirosis involvement in MeN.

Signs of chronic glomerular ischemia with wrinkling of the glomerular capillaries and/or thickening of Bowman´s capsule were found in seven of the included patients. Chronic glomerular ischemia is often found in ageing and hypertensive kidney disease where intimal thickening of renal arteries are apparent [52, 53]. In our study, three patient had moderate intimal thickening in arteries that might explain the glomerular ischemia, but the remaining four had only mild or no intimal thickening, suggesting that other etiologies are responsible for these glomerular lesions. Similar findings with glomerular ischemia not caused by arterial pathology was found in nearly all patients in our previous studies in Central America [6, 7].

The kidney morphology in the present study does not resemble Aristolochic acid nephropathy, an endemic nephropathy described in the Balkans [54]. Aristolochic acid nephropathy typically shows a widespread tubulointerstitial disease but normal glomeruli and the disease is correlated to an increased risk of urothelial cancer [55], something that has not been reported in CKDu patients.

In MeN studies from Central America, low serum sodium, potassium and magnesium levels have been common features [6, 7]. In the present study, this was also found but less pronounced. However, some patients in this cohort were taking medications that could influence electrolyte levels (Table 2). In the two patients with hypokalemia, urine potassium levels indicated renal potassium wasting, similar to what we found in MeN patients [7]. Low serum magnesium was common in this cohort, and interestingly, the FEMg was elevated in all patients, indicating renal magnesium losses. However, in CKD patients, FEMg may be increased to prevent hypermagnesemia [56], but this compensatory mechanism unlikely explains the high FEMg found in the patients with hypomagnesemia in the present study. Hypermagnesuria and hyperkaluria have also been reported as a frequent finding in patients with MeN [57], which supports that the diseases are similar. We suggest that the low serum levels of electrolytes are secondary to renal wasting of electrolytes, probably caused by chronic tubular damage. Further, high renal output of electrolytes will make patients more susceptible to salt depletion and/or dehydration due to sweating in hot climates, which could aggravate already-existing kidney damage. A more in-depth analysis of the urine composition should be studied in larger cohorts.

Heat stress with repeated volume and salt depletion has been proposed to be a main factor behind MeN in Central America [58, 59], a disease that often affects sugarcane workers. However, while the sugarcane workers in Central America experience a high level of heat stress working in the plantations without shade during a long harvest period, the participants in this study were self-employed farmers working in the rice fields approximately 2–3 weeks per half-year and had access to shading trees during breaks. Even though the occupational heat exposures differ, both regions have a hot climate and the possibility for heat stress as a contributing factor in CKDu in Sri Lanka should be further studied.

Uric acid has been suggested to be of significance in the pathogenesis of MeN in Central America, since uric acid crystals have been found in the urine of some sugarcane workers during harvest [59, 60]. In our study we did not find any crystals in the urine and urine levels of uric acid were normal. However the samples in this study were not collected during work days making a comparison difficult.

Heavy metal exposure has been suggested as a possible etiology behind the CKDu endemic in Sri Lanka. In this study, urine measurements of six heavy metals all showed non-toxic levels, thus not supporting a direct nephrotoxic effect of heavy metals. However, one must take into consideration that urinary heavy metal levels mostly mirrors recent exposure, except for urinary cadmium, which has been found to correlate with the accumulation of cadmium in the kidney [34, 61].

In summary, we found that the morphology and biochemical characteristics of CKDu patients in Sri Lanka have many resemblances with the CKDu epidemic in Central America, indicating a similar diagnostic entity. The search for common etiologies affecting both regions should be further studied. However, the mixed morphology pattern seen in Sri Lanka calls for larger biopsy studies with a focus on different possible etiologies affecting renal function in Sri Lanka, such as possible renal-damaging infections. Regarding future studies, we believe that studies of gene and protein expression in biopsy material would give important knowledge about possible etiologies and pathophysiological pathways in the CKDu endemics affecting both Sri Lanka and Central America.

## Supporting information

S1 Table Questionnaire results of self-reported chemical exposure to pesticides and fertilizers. N = 11.
(DOCX)

S2 Table Supporting data set. (DOCX)

## References

[1] Jha V, Garcia-Garcia G, Iseki K, Li Z, Naicker S, Plattner B, et al Chronic kidney disease: global dimension and perspectives. Lancet. 2013;382(9888): 260–272. doi: 10.1016/S0140-6736(13)60687-X 23727169 10.1016/S0140-6736(13)60687-X

[2] Weaver VM, Fadrowski JJ, Jaar BG. Global dimensions of chronic kidney disease of unknown etiology (CKDu): a modern era environmental and/or occupational nephropathy? BMC Nephrol. 2015;16: 145 doi: 10.1186/s12882-015-0105-6 26282933 10.1186/s12882-015-0105-6 PMC4539684

[3] Bello AK, Levin A, Tonelli M, Okpechi IG, Feehally J, Harris D, et al Assessment of Global Kidney Health Care Status. JAMA. 2017;317(18): 1864–1881. doi: 10.1001/jama.2017.4046 28430830 10.1001/jama.2017.4046 PMC5470418

[4] Weiner DE, McClean MD, Kaufman JS, Brooks DR. The Central American epidemic of CKD. Clin J Am Soc Nephrol. 2013;8(3): 504–511. doi: 10.2215/CJN.05050512 23099656 10.2215/CJN.05050512

[5] UpToDate. Mesoamerican nephropathy. http://www.uptodate.com/contents/mesoamerican-nephropathy.

[6] Wijkstrom J, Leiva R, Elinder CG, Leiva S, Trujillo Z, Trujillo L, et al Clinical and pathological characterization of Mesoamerican nephropathy: a new kidney disease in Central America. Am J Kidney Dis. 2013;62(5): 908–918. doi: 10.1053/j.ajkd.2013.05.019 23850447 10.1053/j.ajkd.2013.05.019

[7] Wijkstrom J, Gonzalez-Quiroz M, Hernandez M, Trujillo Z, Hultenby K, Ring A, et al Renal Morphology, Clinical Findings, and Progression Rate in Mesoamerican Nephropathy. Am J Kidney Dis. 2017;69(5): 626–636. doi: 10.1053/j.ajkd.2016.10.036 28126239 10.1053/j.ajkd.2016.10.036

[8] Wegman D CJ, Hogstedt C, Jakobsson K, Wesseling C. Mesoamerican nephropathy: report from the second international research workshop on MeN. Heredia, Costa Rica: SALTRA / IRET- UNA; 2016. http://repositorio.una.ac.cr/bitstream/handle/11056/13142/MeN%202015%20Scientific%20Report%20high%20resolution_corregida.pdf?sequence=3&isAllowed=y.

[9] WHO. Country statistics and global health estimates by WHO and UN partners—Sri Lanka; 2012. http://www.who.int/gho/countries/lka.pdf?ua=1.

[10] Athuraliya TN, Abeysekera DT, Amerasinghe PH, Kumarasiri PV, Dissanayake V. Prevalence of chronic kidney disease in two tertiary care hospitals: high proportion of cases with uncertain aetiology. Ceylon Med J. 2009;54(1): 23–25. 19391454 10.4038/cmj.v54i1.471

[11] Athuraliya NT, Abeysekera TD, Amerasinghe PH, Kumarasiri R, Bandara P, Karunaratne U, et al Uncertain etiologies of proteinuric-chronic kidney disease in rural Sri Lanka. Kidney Int. 2011;80(11): 1212–1221. doi: 10.1038/ki.2011.258 21832982 10.1038/ki.2011.258

[12] Wanigasuriya K. Update on uncertain etiology of chronic kidney disease in Sri Lanka’s north-central dry zone. MEDICC Rev. 2014;16(2): 61–65. 24878651 10.37757/MR2014.V16.N2.10

[13] Jayatilake N, Mendis S, Maheepala P, Mehta FR, Team CKNRP. Chronic kidney disease of uncertain aetiology: prevalence and causative factors in a developing country. BMC Nephrol. 2013;14: 180 doi: 10.1186/1471-2369-14-180 23981540 10.1186/1471-2369-14-180 PMC3765913

[14] Chandrajith R, Nanayakkara S, Itai K, Aturaliya TN, Dissanayake CB, Abeysekera T, et al Chronic kidney diseases of uncertain etiology (CKDue) in Sri Lanka: geographic distribution and environmental implications. Environ Geochem Health. 2011;33(3): 267–278. doi: 10.1007/s10653-010-9339-1 20853020 10.1007/s10653-010-9339-1

[15] Jayasekara KB, Dissanayake DM, Sivakanesan R, Ranasinghe A, Karunarathna RH, Priyantha Kumara GW. Epidemiology of chronic kidney disease, with special emphasis on chronic kidney disease of uncertain etiology, in the north central region of Sri Lanka. J Epidemiol. 2015;25(4): 275–280. doi: 10.2188/jea.JE20140074 25787679 10.2188/jea.JE20140074 PMC4375281

[16] Jayasumana C, Paranagama P, Agampodi S, Wijewardane C, Gunatilake S, Siribaddana S. Drinking well water and occupational exposure to Herbicides is associated with chronic kidney disease, in Padavi-Sripura, Sri Lanka. Environ Health. 2015;14(1): 6. 25596925 10.1186/1476-069X-14-6 PMC4417209

[17] Wanigasuriya KP, Peiris-John RJ, Wickremasinghe R, Hittarage A. Chronic renal failure in North Central Province of Sri Lanka: an environmentally induced disease. Trans R Soc Trop Med Hyg. 2007;101(10): 1013–1017. doi: 10.1016/j.trstmh.2007.05.006 17643458 10.1016/j.trstmh.2007.05.006

[18] De Silva P, Abdul KSM, Eakanayake E, Jayasinghe SS, Jayasumana C, Asanthi HB, et al Urinary Biomarkers KIM-1 and NGAL for Detection of Chronic Kidney Disease of Uncertain Etiology (CKDu) among Agricultural Communities in Sri Lanka. PLoS Negl Trop Dis. 2016;10(9): 17. 10.1371/journal.pntd.0004979 PMC5028052 27643785

[19] Nanayakkara S, Senevirathna ST, Karunaratne U, Chandrajith R, Harada KH, Hitomi T, et al Evidence of tubular damage in the very early stage of chronic kidney disease of uncertain etiology in the North Central Province of Sri Lanka: a cross-sectional study. Environ Health Prev Med. 2012;17(2): 109–117. doi: 10.1007/s12199-011-0224-z 21710150 10.1007/s12199-011-0224-z PMC3342629

[20] Nanayakkara S, Komiya T, Ratnatunga N, Senevirathna ST, Harada KH, Hitomi T, et al Tubulointerstitial damage as the major pathological lesion in endemic chronic kidney disease among farmers in North Central Province of Sri Lanka. Environ Health Prev Med. 2012;17(3): 213–221. doi: 10.1007/s12199-011-0243-9 21993948 10.1007/s12199-011-0243-9 PMC3348245

[21] Wijetunge S, Ratnatunga NV, Abeysekera TD, Wazil AW, Selvarajah M. Endemic chronic kidney disease of unknown etiology in Sri Lanka: Correlation of pathology with clinical stages. Indian J Nephrol. 2015;25(5): 274–280. doi: 10.4103/0971-4065.145095 26628792 10.4103/0971-4065.145095 PMC4588322

[22] Badurdeen Z, Nanayakkara N, Ratnatunga NV, Wazil AW, Abeysekera TD, Rajakrishna PN, et al Chronic kidney disease of uncertain etiology in Sri Lanka is a possible sequel of interstitial nephritis! Clin Nephrol. 2016;86 (2016)(13): 106–109. doi: 10.5414/CNP86S115 27469156 10.5414/CNP86S115

[23] Selvarajah M, Weeratunga P, Sivayoganthan S, Rathnatunga N, Rajapakse S. Clinicopathological correlates of chronic kidney disease of unknown etiology in Sri Lanka. Indian J Nephrol. 2016;26(5): 357–363. doi: 10.4103/0971-4065.167280 27795631 10.4103/0971-4065.167280 PMC5015515

[24] Jayasumana C, Gunatilake S, Siribaddana S. Simultaneous exposure to multiple heavy metals and glyphosate may contribute to Sri Lankan agricultural nephropathy. BMC Nephrol. 2015;16: 103 doi: 10.1186/s12882-015-0109-2 26162605 10.1186/s12882-015-0109-2 PMC4499177

[25] Bandara JM, Senevirathna DM, Dasanayake DM, Herath V, Bandara JM, Abeysekara T, et al Chronic renal failure among farm families in cascade irrigation systems in Sri Lanka associated with elevated dietary cadmium levels in rice and freshwater fish (Tilapia). Environ Geochem Health. 2008;30(5): 465–478. doi: 10.1007/s10653-007-9129-6 18200439 10.1007/s10653-007-9129-6

[26] Gamage CD, Yoshimatsu K, Sarathkumara YD, Kulendiran T, Nanayakkara N, Arikawa J. Serological evidence of hantavirus infection in Girandurukotte, an area endemic for chronic kidney disease of unknown aetiology (CKDu) in Sri Lanka. Int J Infect Dis. 2017;57: 77–78. doi: 10.1016/j.ijid.2017.02.004 28212862 10.1016/j.ijid.2017.02.004

[27] Bandara JM, Wijewardena HV, Liyanege J, Upul MA, Bandara JM. Chronic renal failure in Sri Lanka caused by elevated dietary cadmium: Trojan horse of the green revolution. Toxicol Lett. 2010;198(1): 33–39. doi: 10.1016/j.toxlet.2010.04.016 20430069 10.1016/j.toxlet.2010.04.016 PMC7127468

[28] Racusen LC, Solez K, Colvin RB, Bonsib SM, Castro MC, Cavallo T, et al The Banff 97 working classification of renal allograft pathology. Kidney Int. 1999;55(2): 713–723. doi: 10.1046/j.1523-1755.1999.00299.x 9987096 10.1046/j.1523-1755.1999.00299.x

[29] Levey AS, Stevens LA, Schmid CH, Zhang YL, Castro AF 3rd, Feldman HI, et al A new equation to estimate glomerular filtration rate. Ann Intern Med. 2009;150(9): 604–612. 19414839 10.7326/0003-4819-150-9-200905050-00006 PMC2763564

[30] Inker LA, Schmid CH, Tighiouart H, Eckfeldt JH, Feldman HI, Greene T, et al Estimating glomerular filtration rate from serum creatinine and cystatin C. N Engl J Med. 2012;367(1): 20–29. doi: 10.1056/NEJMoa1114248 22762315 10.1056/NEJMoa1114248 PMC4398023

[31] United States Environmental Protection Agency, EPA. Method 200.8. Determination of Trace Elements in Waters and Wastes by Inductively Coupled Plasma-Mass Spectrometry; 1994. https://www.epa.gov/sites/production/files/2015-06/documents/epa-200.8.pdf.

[32] Agency for Toxic Substances and Disease Registry. ToxGuide™ for Arsenic. 2007. http://www.atsdr.cdc.gov/toxguides/toxguide-2.pdf. 37184170

[33] Roels HA, Hoet P, Lison D. Usefulness of biomarkers of exposure to inorganic mercury, lead, or cadmium in controlling occupational and environmental risks of nephrotoxicity. Ren Fail. 1999;21(3–4): 251–262. doi: 10.3109/08860229909085087 10416202 10.3109/08860229909085087

[34] Bernard A. Renal dysfunction induced by cadmium: biomarkers of critical effects. Biometals. 2004;17(5): 519–523. 15688856 10.1023/b:biom.0000045731.75602.b9

[35] Sommar JN, Hedmer M, Lundh T, Nilsson L, Skerfving S, Bergdahl IA. Investigation of lead concentrations in whole blood, plasma and urine as biomarkers for biological monitoring of lead exposure. J Expo Sci Environ Epidemiol. 2014;24(1): 51–57. doi: 10.1038/jes.2013.4 23443239 10.1038/jes.2013.4

[36] Paschal DC, Ting BG, Morrow JC, Pirkle JL, Jackson RJ, Sampson EJ, et al Trace metals in urine of United States residents: reference range concentrations. Environ Res. 1998;76(1): 53–59. doi: 10.1006/enrs.1997.3793 9466897 10.1006/enrs.1997.3793

[37] Agency for Toxic Substances and Disease Registry. ToxGuide^™^ for Uranium. 2013. https://www.atsdr.cdc.gov/toxguides/toxguide-150.pdf. 24049861

[38] Agency for Toxic Substances and Disease Registry. ToxGuide™ for Vanadium. 2012. https://www.atsdr.cdc.gov/toxguides/toxguide-58.pdf. 37262203

[39] UpToDate. Evaluation of the adult patient with hypokalemia. http://www.uptodate.com/contents/evaluation-of-the-adult-patient-with-hypokalemia.

[40] Elisaf M, Siamopoulos KC. FRACTIONAL EXCRETION OF POTASSIUM IN NORMAL SUBJECTS AND IN PATIENTS WITH HYPOKALEMIA. Postgrad Med J. 1995;71(834): 211–212. 7784279 10.1136/pgmj.71.834.211 PMC2398075

[41] Elisaf M, Panteli K, Theodorou J, Siamopoulos KC. Fractional excretion of magnesium in normal subjects and in patients with hypomagnesemia. Magnes Res. 1997;10(4): 315–320. 9513927

[42] Jarup L, Hellstrom L, Alfven T, Carlsson MD, Grubb A, Persson B, et al Low level exposure to cadmium and early kidney damage: the OSCAR study. Occup Environ Med. 2000;57(10): 668–672. doi: 10.1136/oem.57.10.668 10984338 10.1136/oem.57.10.668 PMC1739874

[43] Castano A, Sanchez-Rodriguez JE, Canas A, Esteban M, Navarro C, Rodriguez-Garcia AC, et al Mercury, lead and cadmium levels in the urine of 170 Spanish adults: A pilot human biomonitoring study. Int J Hyg Environ Health. 2012;215(2): 191–195. doi: 10.1016/j.ijheh.2011.09.001 21968334 10.1016/j.ijheh.2011.09.001

[44] Caldwell KL, Jones RL, Verdon CP, Jarrett JM, Caudill SP, Osterloh JD. Levels of urinary total and speciated arsenic in the US population: National Health and Nutrition Examination Survey 2003–2004. J Expo Sci Environ Epidemiol. 2009;19(1): 59–68. doi: 10.1038/jes.2008.32 18523458 10.1038/jes.2008.32

[45] Lee JW, Lee CK, Moon CS, Choi IJ, Lee KJ, Yi SM, et al Korea National Survey for Environmental Pollutants in the Human Body 2008: Heavy metals in the blood or urine of the Korean population. Int J Hyg Environ Health. 2012;215(4): 449–457. doi: 10.1016/j.ijheh.2012.01.002 22341685 10.1016/j.ijheh.2012.01.002

[46] Fischer RSB, Vangala C, Truong L, Mandayam S, Chavarria D, Granera Llanes OM, et al Early detection of acute tubulointerstitial nephritis in the genesis of Mesoamerican nephropathy. Kidney Int. international. Forthcoming 2017. 10.1016/j.kint.2017.09.012 29162294

[47] George J, Patnaik M, Bakshi E, Levy Y, Ben-David A, Ahmed A, et al Hantavirus seropositivity in Israeli patients with renal failure. Viral Immunol. 1998;11(2): 103–108. doi: 10.1089/vim.1998.11.103 9765032 10.1089/vim.1998.11.103

[48] Peter JB, Patnaik M, Gott P, Weins B, Souw PT. Antibodies to different strains of hantavirus in end-stage renal disease in USA and Japan. Lancet. 1994;343(8890): 181. 10.1016/s0140-6736(94)90976-8 7904033

[49] Sunil-Chandra NP, Clement J, Maes P, DES HJ, VANE M, VANR M. Concomitant leptospirosis-hantavirus co-infection in acute patients hospitalized in Sri Lanka: implications for a potentially worldwide underestimated problem. Epidemiol Infect. 2015;143(10): 2081–2093. doi: 10.1017/S0950268814003707 25582980 10.1017/S0950268814003707 PMC9506992

[50] Murray KO, Fischer RS, Chavarria D, Duttmann C, Garcia MN, Gorchakov R, et al Mesoamerican nephropathy: a neglected tropical disease with an infectious etiology? Microbes Infect. 2015;17(10): 671–675. doi: 10.1016/j.micinf.2015.08.005 26320026 10.1016/j.micinf.2015.08.005

[51] Hotez PJ, Woc-Colburn L, Bottazzi ME. Neglected tropical diseases in Central America and Panama: Review of their prevalence, populations at risk and impact on regional development. Int J Parasit. 2014;44(9): 597–603. 10.1016/j.ijpara.2014.04.001 24846528

[52] Glassock RJ, Rule AD. The implications of anatomical and functional changes of the aging kidney: with an emphasis on the glomeruli. Kidney Int. 2012;82(3): 270–277. doi: 10.1038/ki.2012.65 22437416 10.1038/ki.2012.65 PMC3513938

[53] Colvin RB. Diagnostic pathology Kidney diseases. 1st ed Salt Lake City, Utah: Amirsys; 2011.

[54] Gokmen MR, Lord GM. Aristolochic acid nephropathy. BMJ. 2012;344:e4000 doi: 10.1136/bmj.e4000 22705815 10.1136/bmj.e4000

[55] Gokmen MR, Cosyns JP, Arlt VM, Stiborova M, Phillips DH, Schmeiser HH, et al The Epidemiology, Diagnosis, and Management of Aristolochic Acid Nephropathy A Narrative Review. Ann Intern Med. 2013;158(6): 469–U132. doi: 10.7326/0003-4819-158-6-201303190-00006 23552405 10.7326/0003-4819-158-6-201303190-00006

[56] Cunningham J, Rodriguez M, Messa P. Magnesium in chronic kidney disease Stages 3 and 4 and in dialysis patients. Clin Kidney J. 2012;5(Suppl 1): i39–i51. doi: 10.1093/ndtplus/sfr166 26069820 10.1093/ndtplus/sfr166 PMC4455820

[57] Herrera R, Orantes CM, Almaguer M, Alfonso P, Bayarre HD, Leiva IM, et al Clinical characteristics of chronic kidney disease of nontraditional causes in Salvadoran farming communities. MEDICC Rev. 2014;16(2): 39–48. 24878648 10.37757/MR2014.V16.N2.7

[58] Wesseling C, Aragon A, Gonzalez M, Weiss I, Glaser J, Bobadilla NA, et al Kidney function in sugarcane cutters in Nicaragua—A longitudinal study of workers at risk of Mesoamerican nephropathy. Environ Res. 2016;147: 125–132. doi: 10.1016/j.envres.2016.02.002 26866450 10.1016/j.envres.2016.02.002

[59] Garcia-Trabanino R, Jarquin E, Wesseling C, Johnson RJ, Gonzalez-Quiroz M, Weiss I, et al Heat stress, dehydration, and kidney function in sugarcane cutters in El Salvador—A cross-shift study of workers at risk of Mesoamerican nephropathy. Environ Res. 2015;142: 746–755. doi: 10.1016/j.envres.2015.07.007 26209462 10.1016/j.envres.2015.07.007

[60] Roncal-Jimenez C, Garcia-Trabanino R, Barregard L, Lanaspa MA, Wesseling C, Harra T, et al Heat Stress Nephropathy From Exercise-Induced Uric Acid Crystalluria: A Perspective on Mesoamerican Nephropathy. Am J Kidney Dis. 2016;67(1): 20–30. doi: 10.1053/j.ajkd.2015.08.021 26455995 10.1053/j.ajkd.2015.08.021

[61] Akerstrom M, Barregard L, Lundh T, Sallsten G. The relationship between cadmium in kidney and cadmium in urine and blood in an environmentally exposed population. Toxicol Appl Pharmacol. 2013;268(3): 286–293. doi: 10.1016/j.taap.2013.02.009 23454399 10.1016/j.taap.2013.02.009

