# Supplementary material for: Morphological and clinical findings in Sri Lankan patients with chronic kidney disease of unknown cause (CKDu): Similarities and differences with Mesoamerican Nephropathy
Source: PLoS One. 2018 Mar 7;13(3):e0193056. doi: 10.1371/journal.pone.0193056 (PMC5841753; doi:10.1371/journal.pone.0193056)
Supplement: S1 Table — N = 11. (DOCX) [file pone.0193056.s001.docx]

|  | Cases (percentage) | Type of chemical |
| --- | --- | --- |
| Urea | 11 (100%) | Fertilizer |
| TSP-triple super phosphate | 11 (100%) | Fertilizer |
| MoP-Muriate of Potash or potassium chloride | 11 (100%) | Fertilizer |
| Glyphosate | 10 (91%) | Herbicide |
| MCPA  (2-methyl-4-chlorophenoxyacetic acid) | 5 (45%) | Herbicide |
| Chlorpyrifos | 5 (45%) | Insecticide |
| Pretilachlor | 4 (36%) | Herbicide |
| Paraquat | 3 (27%) | Herbicide |
| Carbofuran | 3 (27%) | Insecticide |
| DCMU (3-(3,4-dichlorophenyl)-1,1-dimethylurea | 3 (27%) | Herbicide |
| Propanil | 2 (18%) | Herbicide |
| Diazinon | 2 (18%) | Insecticide |
| Bispyribac-sodium | 2 (18%) | Herbicide |
| Fenoxaprop-p-ethyl | 2 (18%) | Fungicide, Herbicide |
| Mancozeb | 1 (9%) | Fungicide |
| Sofit (Pretilachlor + CGA 123'407) | 1 (9%) | Herbicide |
