# Supplementary material for: Morphological and clinical findings in Sri Lankan patients with chronic kidney disease of unknown cause (CKDu): Similarities and differences with Mesoamerican Nephropathy
Source: PLoS One. 2018 Mar 7;13(3):e0193056. doi: 10.1371/journal.pone.0193056 (PMC5841753; doi:10.1371/journal.pone.0193056)
Supplement: S2 Table — (DOCX) [file pone.0193056.s002.docx]

| Patient study number | 1 | 2 | 3 | 4 | 5 | 6 | 7 | 8 | 9 | 10 | 11 |
| --- | --- | --- | --- | --- | --- | --- | --- | --- | --- | --- | --- |
| BMI (kg/m²) | 21 | 28 | 19 | 23 | 19 | 16 | 21 | 21 | 19 | 19 | 19 |
| Systolic BP (mmHg) | 132 | 97 | 120 | 145 | 120 | 160 | 100 | 100 | 110 | 130 | 130 |
| Diastolic BP (mmHg) | 86 | 58 | 80 | 90 | 80 | 80 | 70 | 70 | 80 | 80 | 80 |
| Kidney length (cm) | 8.2 | 9.8 | 8 | 8.8 | 8.8 | 9 | 7.8 | 8.1 | 9.2 | 8.8 | 9.1 |
| Total farming years | 40 | 20 | 25 | 15 | 40 | 20 | 34 | 40 | 7 | 17 | 3 |
| Days working in field/year | 20 | 20 | 28 | 20 | 25 | 25 | 40 | 50 | 40 | 80 | 58 |
| Total days working in fields | 800 | 400 | 700 | 300 | 1000 | 500 | 1360 | 2000 | 280 | 1360 | 174 |
| Smoking years | 5 | 3 | 25 | 6 | 30 | 0 | 25 | 0 | 4 | 0 | 20 |
| Pack years | 1.2 | 2.2 | 0.1 | 1.5 | 3.0 | 0.0 | 6.2 | 0.0 | 0.8 | 0.0 | 2.0 |
| Liquid intake/day (L) | 5 | 5 | 4 | 4 | 3 | 4.5 | 5 | 3 | 4 | 6 | 3.5 |
| % water | 81% | 86% | 85% | 74% | 78% | 91% | 80% | 93% | 84% | 97% | 80% |
| % tea | 18% | 12% | 15% | 25% | 20% | 9% | 20% | 7% | 15% | 3% | 20% |
| Herbal medicine prior use | yes | yes | yes | yes | yes | no | yes | no | no | no | yes |
| Type of herbal medicine | Ayurveda | Ayurveda | Ayurveda | Ayurveda | Ayurveda | - | Ayurveda | - | - | - | Ayurveda |
| Herbal medicine duration (years) | 0.5 | 2 | 0.5 | 0.08 | 0.08 | - | 0.08 | - | - | - | 0.17 |
| Herbal medicine current use | no | no | no | no | no | no | no | no | no | no | no |
|  |  |  |  |  |  |  |  |  |  |  |  |
| Serum/Blood |  |  |  |  |  |  |  |  |  |  |  |
| Creatinine (µmol/L) | 120 | 168 | 267 | 172 | 182 | 163 | 150 | 262 | 121 | 189 | 227 |
| Cystatin C (mg/L) | 1.18 | 2.07 | 2.67 | 2.15 | 2.12 | 1.54 | 1.8 | 2.9 | 1.27 | 2.03 | 1.83 |
| eGFRcr (ml/min/1.73m²) | 56 | 40 | 21 | 40 | 35 | 45 | 45 | 22 | 70 | 39 | 29 |
| eGFRcr+cyst c (ml/min/1.73m²) | 59 | 34 | 20 | 33 | 31 | 46 | 40 | 20 | 66 | 35 | 32 |
| Sodium (mmol/L) | 147 | 140 | 144 | 143 | 139 | 138 | 139 | 134 | 142 | 140 | 137 |
| Potassium (mmol/L) | 2.2 | 4.7 | 3.4 | 4.5 | 6.3 | 4.1 | 4.3 | 4.3 | 4.3 | 4.7 | 4.3 |
| Magnesium (mmol/L) | 0.43 | 0.61 | 0.86 | 0.81 | 0.44 | 0.79 | 0.64 | 0.71 | 0.76 | 0.8 | 0.88 |
| Uric Acid (µmol/L) | 201 | 413 | 480 | 362 | 583 | 453 | 406 | 489 | 351 | 465 | 418 |
| Calcium (mmol/L) | 1.12 | 2.37 | 2.3 | 2.44 | 2.07 | 2.56 | 2.18 | 2.3 | 2.55 | 2.5 | 2.37 |
| Albumin (g/L) | 18 | 37 | 37 | 43 | 40 | 45 | 34 | 36 | 44 | 41 | 39 |
| Phosphate (mmol/L) | 0.59 | 1.2 | 0.88 | 1.4 | 1 | 1.2 | 1 | 1.1 | 0.94 | 0.81 | 0.7 |
| Glucose (mmol/L) | 2.9 | 6.8 | 5.4 | 5.3 | 5.2 | 5.3 | 3.7 | 6 | 4.8 | 4.9 | 6.4 |
| Hemoglobin (g/L) | 156 | 144 | 103 | 105 | 140 | 129 | 117 | 100 | 135 | 129 | 102 |
| White blood cell count (10⁹/L) | 7.1 | 7.13 | 7.9 | 7.32 | 7.76 | 6.97 | 4.91 | 8.35 | 5.85 | 7.29 | 10.25 |
| Aldosterone (nmol/L) | 0.771 | 0.094 | 0.465 | 0.241 | 0.357 | 0.366 | 0.176 | 0.953 | 0.23 | 0.174 | 0.177 |
| Renin (mIU/L) | 90 | 10 | 36 | 3.1 | 50 | 33 | 13 | 40 | 15 | 11 | 3.1 |
|  |  |  |  |  |  |  |  |  |  |  |  |
| Urine |  |  |  |  |  |  |  |  |  |  |  |
| Creatinine (mmol/L) | 10.1 | 17.2 | 8.2 | 12.7 | 4 | 11.6 | 9.2 | 8.7 | 6 | 2.6 | 2.4 |
| ACR (mg/mmol) | 11.3 | 0.1 | 0.2 | 1.1 | 13.0 | 4.9 | 0.2 | 47.2 | 0.3 | 0.6 | 0.6 |
| A1M-creatinine ratio (mg/mmol) | 14.5 | 5.1 | 6.5 | 0.8 | 5.6 | 4.0 | 1.9 | 10.0 | 1.9 | 1.2 | 6.0 |
| NGAL (µg/L) | 111 | <35 | 154 | <35 | <35 | <35 | <35 | 115 | <35 | <35 | 44 |
| KIM-1 (ng/ml) | 0.83 | 2.20 | 0.66 | 0.19 | 1.30 | 0.35 | 3.44 | 1.44 | 0.37 | 0.30 | 1.07 |
| Sodium (mmol/L) | 63 | 299 | 120 | 75 | 68 | 116 | 106 | 100 | 113 | 36 | 48 |
| FENa (%) | 0.5 | 2.1 | 2.7 | 0.7 | 2.2 | 1.2 | 1.2 | 2.2 | 1.6 | 1.9 | 3.3 |
| Potassium (mmol/L) | 46 | 50 | 24 | 38 | 21 | 31 | 29 | 29 | 22 | 5 | 7 |
| Potassium-creatinine ratio | 4.6 | 2.9 | 2.9 | 3.0 | 5.3 | 2.7 | 3.2 | 3.3 | 3.7 | 1.9 | 2.9 |
| FEK (%) | 25 | 10 | 23 | 11 | 15 | 11 | 11 | 20 | 10 | 8 | 15 |
| Magnesium (mmol/L) | 1.9 | 3.7 | 3.2 | 2.7 | 1.1 | 1.6 | 2.6 | 2.0 | 1.7 | 1.1 | 1.1 |
| FEMg (%) | 7 | 8 | 17 | 6 | 16 | 4 | 9 | 12 | 6 | 14 | 17 |
| Uric acid (mmol/L) | 1.7 | 2.6 | 1.4 | 1.5 | 0.9 | 2 | 1.3 | 1.1 | 1.1 | 0.5 | 0.5 |
| Uric acid-creatinine ratio | 0.17 | 0.15 | 0.17 | 0.12 | 0.23 | 0.17 | 0.14 | 0.13 | 0.18 | 0.19 | 0.21 |
| Arsenic (µg/L) | 24.1 | 77 | 32 | 26.9 | 44.4 | 48.9 | 46.6 | 56.7 | 38.8 | 9.88 | 4.54 |
| Arsenic-creatinine ratio (µg/g) | 21 | 40 | 34 | 19 | 98 | 37 | 45 | 58 | 57 | 34 | 17 |
| Cadmium (µg/L) | 1.100 | 0.873 | 0.462 | 0.282 | 0.223 | 0.282 | 0.342 | 0.412 | 0.302 | 0.108 | 0.284 |
| Cadmium-creatinine ratio (µg/g) | 0.96 | 0.45 | 0.50 | 0.20 | 0.49 | 0.21 | 0.33 | 0.42 | 0.44 | 0.37 | 1.05 |
| Mercury (µg/L) | 0.238 | 0.689 | 0.26 | 0.212 | 0.358 | <0.2 | <0.2 | <0.2 | 0.394 | <0.2 | <0.2 |
| Mercury-creatinine ratio (µg/g) | 0.21 | 0.35 | 0.28 | 0.15 | 0.79 | 0.08 | 0.10 | 0.10 | 0.58 | 0.34 | 0.37 |
| Lead (µg/L) | 0.948 | 1.29 | 1.72 | 0.664 | <0.5 | <0.5 | 0.515 | <0.5 | <0.5 | <0.5 | <0.5 |
| Uranium (µg/L) | <0.02 | <0.02 | <0.02 | <0.02 | <0.02 | <0.02 | <0.02 | <0.02 | <0.02 | <0.02 | <0.02 |
| Vanadium (µg/L) | <0.1 | <0.1 | <0.1 | <0.1 | <0.1 | 0.146 | 0.125 | <0.1 | <0.1 | <0.1 | 0.221 |
|  |  |  |  |  |  |  |  |  |  |  |  |
| Kidney biopsy morphology |  |  |  |  |  |  |  |  |  |  |  |
| Total number of glomeruli | 7 | 13 | 20 | 20 | 14 | 13 | 29 | 14 | 14 | 15 | 24 |
| % globally sclerosed glomeruli | 29 | 54 | 75 | 45 | 64 | 54 | 41 | 57 | 29 | 20 | 8 |
| Glomerular size (0-3) | 1 | 2 | 1 | 1 | 2 | 2 | 2 | 3 | 1 | 1 | 1 |
| Wrinkled GBM/periglom fibrosis | 0 | 0 | 1 | 0 | 1 | 1 | 1 | 1 | 0 | 1 | 1 |
| Tubular atrophy (0-3) | 1 | 1 | 2 | 1 | 1 | 1 | 1 | 1 | 1 | 1 | 1 |
| Interstitial fibrosis (0-3) D/F | 1F | 1F | 3D | 1F | 2F | 1F | 2D | 2D | 1F | 1F | 2D |
| Interstitial inflammation (0-3) | 0 | 1 | 3 | 1 | 1 | 1 | 2 | 1 | 0 | 3 | 2 |
| Intimal thickening (0-3) | - | 0 | 2 | 0 | 2 | 0 | 0 | 2 | 0 | 1 | 1 |
| Smooth muscle hyperplasia (0-3) | - | 0 | 1 | 0 | 1 | 0 | 1 | 0 | 0 | 1 | 0 |
| Arteriolar hyalinosis (0-3) | 1 | 1 | 1 | 2 | 1 | 2 | 1 | 1 | 0 | 1 | 2 |
| GBM thickness (nm) | 349 | 347 | 259 | 260 | 337 | 263 | 276 | 346 | 282 | 345 | 309 |
| Podocyte foot processes (slits/µm GBM) | 1.7 | 1.4 | 1.4 | 1.3 | 1.8 | 1.3 | 1.7 | 1.4 | 1.6 | 1.5 | 1.2 |
